# Risk of Typical Diabetes-Associated Complications in Different Clusters of Diabetic Patients: Analysis of Nine Risk Factors

**DOI:** 10.3390/jpm11050328

**Published:** 2021-04-22

**Authors:** Michael Leutner, Nils Haug, Luise Bellach, Elma Dervic, Alexander Kautzky, Peter Klimek, Alexandra Kautzky-Willer

**Affiliations:** 1 Department of Internal Medicine III, Clinical Division of Endocrinology and Metabolism, Medical University of Vienna, Waehringer Guertel 18-20, A-1090 Vienna, Austria; michael.leutner@meduniwien.ac.at (M.L.); lbellach@gmx.at (L.B.); 2 Section for Science of Complex Systems, CeMSIIS, Medical University of Vienna, Spitalgasse 23, A-1090 Vienna, Austria; haug@csh.ac.at (N.H.); elma.dervic@meduniwien.ac.at (E.D.); peter.klimek@meduniwien.ac.at (P.K.); 3 Complexity Science Hub Vienna, Josefstaedter Straße 39, 1080 Vienna, Austria; 4 Department of Psychiatry and Psychotherapy, Medical University of Vienna, Waehringer Guertel 18-20, A-1090 Vienna, Austria; alexander.kautzky@meduniwien.ac.at; 5 Gender Institute, A-3571 Gars am Kamp, Austria

**Keywords:** diabetes mellitus, cluster analyses, risk factors, micro- and macrovascular disease

## Abstract

Objectives: Diabetic patients are often diagnosed with several comorbidities. The aim of the present study was to investigate the relationship between different combinations of risk factors and complications in diabetic patients. Research design and methods: We used a longitudinal, population-wide dataset of patients with hospital diagnoses and identified all patients (*n* = 195,575) receiving a diagnosis of diabetes in the observation period from 2003–2014. We defined nine ICD-10-codes as risk factors and 16 ICD-10 codes as complications. Using a computational algorithm, cohort patients were assigned to clusters based on the risk factors they were diagnosed with. The clusters were defined so that the patients assigned to them developed similar complications. Complication risk was quantified in terms of relative risk (RR) compared with healthy control patients. Results: We identified five clusters associated with an increased risk of complications. A combined diagnosis of arterial hypertension (aHTN) and dyslipidemia was shared by all clusters and expressed a baseline of increased risk. Additional diagnosis of (1) smoking, (2) depression, (3) liver disease, or (4) obesity made up the other four clusters and further increased the risk of complications. Cluster 9 (aHTN, dyslipidemia and depression) represented diabetic patients at high risk of angina pectoris “AP” (RR: 7.35, CI: 6.74–8.01), kidney disease (RR: 3.18, CI: 3.04–3.32), polyneuropathy (RR: 4.80, CI: 4.23–5.45), and stroke (RR: 4.32, CI: 3.95–4.71), whereas cluster 10 (aHTN, dyslipidemia and smoking) identified patients with the highest risk of AP (RR: 10.10, CI: 9.28–10.98), atherosclerosis (RR: 4.07, CI: 3.84–4.31), and loss of extremities (RR: 4.21, CI: 1.5–11.84) compared to the controls. Conclusions: A comorbidity of aHTN and dyslipidemia was shown to be associated with diabetic complications across all risk-clusters. This effect was amplified by a combination with either depression, smoking, obesity, or non-specific liver disease.

## 1. Background

The prevalence of diabetes mellitus is constantly increasing, making this disease a global health problem. Efficient diabetes management is necessary in order to decrease the risk of both micro- and macrovascular complications such as cardiovascular diseases or diabetic nephropathy [1], especially when patients are also diagnosed with other comorbidities such as hypertension or depression [2,3]. Data from large trials conducted in recent decades such as the Collaborative Atorvastatin Diabetes Study (CARDS) or the Action in Diabetes and Vascular disease: preterAx and diamicroN-MR Controlled Evaluation (ADVANCE) have fortified a more generalized approach to the management of diabetics by taking into account additional factors such as cholesterol levels or blood pressure [4,5], or risk factors that are known to increase cardiovascular related mortality rate in diabetics [6]. Accordingly, it has been shown that comorbidities such as dyslipidemia, depression, arterial hypertension, or fatty liver disease predispose diabetic patients to an increased risk of developing serious diabetes-specific complications later in life [7,8,9,10]. Given the wide range of common comorbidities in patients with diabetes that may lead to different trajectories of complications, clustering analyses may be especially useful for treatment stratification and personalization in this collective. Earlier studies that defined clusters of diabetic patients on the basis of parameters related to the etiology of diabetes such as insulin resistance or body mass index (BMI) were able to show that these clusters differentiated the risk of developing various diabetes-specific complications [11,12,13,14,15]. Interestingly, the diabetic cluster defined by insulin resistance as the predominant cause of diabetes showed the greatest risk of progressing to renal complications like chronic kidney disease, macroalbuminuria, or end-stage renal disease within the next decade compared to the other clusters defined by autoimmune diabetes, insulin deficiency diabetes, mild age-related diabetes, and mild obesity-related diabetes [11]. The importance of cluster analyses has also been highlighted by a study of Ryu et al., who developed a screening model including gender-specific characteristics for the estimation of an undiagnosed diabetes mellitus in high-risk patients for development of diabetes mellitus, namely patients with a positive family history of diabetes [16]. Additionally, more recently, Nedyalkova et al. showed that *k*-means clustering to detect clinical variables might be useful to stratify type 2 diabetics into distinct subgroups of risk factors [17].

Nevertheless, many aspects have yet to be explored in the novel field of cluster-based analyses for the risk assessment of diabetes-specific complications. Despite the growing number of studies investigating the effect of single concomitant diseases on diabetic complications such as depression, dyslipidemia, fatty liver disease or arterial hypertension [7,8,9,10], data on pooled comorbidities, and the respective impact on the disease trajectory are sparse. The aim of the present study was to investigate the relationship between different clusters including different combinations of selected diabetes-associated comorbidities such as overweight/obesity, dyslipidemia or arterial hypertension and the occurrence of typical micro- and macrovascular complications.

## 2. Methods

### 2.1. Data

We used a longitudinal, population-wide dataset on hospital stays in Austria spanning the period from 1997 to 2014. For each hospital stay, the dataset records a pseudonym for the patient treated, the date of admission and release, and the main and secondary diagnoses associated with the stay, in terms of level-3 ICD-10 codes. The age and sex of each patient are also available. Since each patient is assigned a consistent pseudonym, it is possible to track the development of the state of a patient’s health throughout the years.

### 2.2. Cohort

The cohort consists of the 195,575 patients who did not receive a hospital diagnosis with ICD-10 code A00–N99 from 1997–2002 and were diagnosed with diabetes mellitus (ICD-10 E10–E14) in the observational period from 2003–2014. Moreover, each patient was at least 60 years old in 2014. The requirement that patients did not receive hospital diagnoses throughout the six years from 1997–2002 justifies our assumption that patients were in good health at the beginning of the observation period. The ratio of males in the cohort was 50,3%. The mean age of cohort patients in 2014 was 74 y (67–82) and 82 y (73–89) for males and females, respectively, the values in brackets denoting lower and upper quartiles. The descriptive characteristics of the study cohort are summarized in Table 1.

### 2.3. Risk Factors and Complications

**Figure 1:**
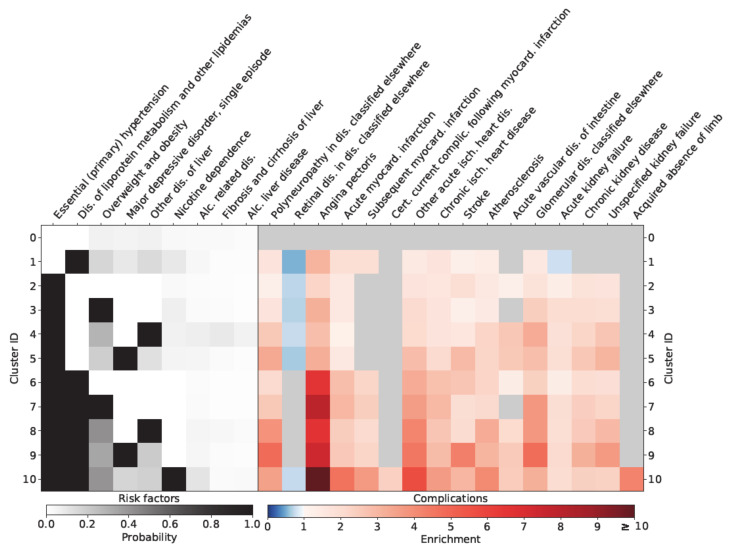
Relationship of different clusters with typical diabetes-specific complications.

We defined nine typical diabetes specific risk factors, and 16 micro and macrovascular complications in terms of level-3 ICD-10 codes. For example, E66 (overweight and obesity) was defined as a risk factor, whereas I64 (stroke) was defined as a complication. The full list of risk factors and complications is given by the labels on the horizontal axis in Figure 1.

### 2.4. Identification of Clusters

The state of each patient’s health is characterized by the set of risk factors and complications which they were diagnosed with during the observation period. Our approach is thus cross-sectional. By using a computational algorithm, we assigned each patient to one of 11 different clusters, each containing more than 5000 individuals. The clusters were constructed algorithmically so that patients in each cluster were diagnosed with combinations of complications during the observation period that were more similar to each other compared to the complications of patients in other clusters. We employed a divisive clustering algorithm that iteratively generates a binary clustering tree, where each node of the tree represents a subset of all patients of the cohort and the leaves of the tree represent the clusters. The result of the algorithm can be read like a decision tree, meaning that each cluster is defined by a set of one or more risk factors diagnosed to each patient in that cluster (inclusion criteria) during the observation period, and a set of risk factors each patient in that cluster was not diagnosed with (exclusion criteria) during that period. It is this property that distinguishes the algorithm from methods as *k*-means [18] or Ward’s method [19], and leads to highly interpretable results. By construction, there exists exactly one cluster without inclusion criteria (the ‘healthy cluster’ or cluster 0). The algorithm aims to maximize the mutual difference of the obtained clusters in terms of the risk of included patients for developing the different complications. A precise definition of the employed algorithm is given in the Supplementary Materials.

We refer the reader to [20,21] for two previous applications of a modified version of the same clustering approach, demonstrating its usefulness in describing health trajectories and its ability to distinguish patient clusters based on diagnoses. It was shown in [22] that in many cases, the underlying clustering algorithm performed similarly well as *k*-means, while offering greater interpretability of its results.

### 2.5. Cluster-Based Relative Risk Analysis

For each cluster *c* and each complication *d*, we computed the relative risk (RR) of a patient in that cluster for receiving a diagnosis of *d* during the observational period compared to patients in the ‘healthy cluster’. This allows us to quantify the extent to which the combination of inclusion criteria of cluster *c* increases the risk of complication *d.* We refer to this fraction as the *enrichment* of complication *d* in cluster *c*. While the clustering procedure is performed with all patients, the cluster-based relative risk analysis is also performed separately for males and females.

A detailed description of the clustering algorithm is given in the Supplementary Materials.

## 3. Results

Figure 1 visualizes the results of the analysis. On the left of the figure, for each cluster *c* and each risk factor *r*, the probability of a patient in that cluster being diagnosed with risk factor *r* is color-coded from white (zero) via gray to black (one). Black signifies that *r* is an inclusion criterion for cluster *c*, meaning that all patients in that cluster have the corresponding diagnosis. For example, depression (F32) and hypertension (I10) are inclusion criteria for cluster 5. The gray color in the boxes corresponding to obesity (E66), recurrent depressive disorder (F33), and unspecific liver disease (K76) also indicate increased probability of these diagnoses compared to the baseline of cluster 0 (the ‘healthy’ cluster). Table 2 gives the demographic characteristics of the different clusters. The clusters with the most unbalanced sex distribution are cluster 10 with more males, and clusters 5 and 9 with more females. Cluster 10 has nicotine dependence as an inclusion criterion, whereas clusters 5 and 9 share major depression as inclusion criterion.

On the right of Figure 1, for each cluster *c* and each complication *d*, the enrichment of *d* in cluster *c* is color-coded, blue shading indicating an enrichment of less than one and red shading denoting an enrichment of more than one. Gray color stands for insignificant results. Significance was tested using a *p*-value of 0.05. Adjustment for multiple hypothesis testing via a Bonferroni correction was found to only bring minor changes to the results and therefore omitted. For example, in cluster 6, the red shading in the box corresponding to angina pectoris (I20) indicates that the risk of patients in cluster 6 of being diagnosed with this condition increases by a factor of around 7 compared to the baseline of the healthy cluster 0. On the other hand, in cluster 5 (aHTN and depression), the light blue shading in the box corresponding to retinal disorders (H36) means that patients in cluster 5 had a slightly lower risk of being diagnosed with H36 compared to the healthy baseline cluster 0.

According to Figure 1, six risk factors were defined as inclusion criteria in at least one cluster, namely obesity, dyslipidemia, smoking, depression, arterial hypertension, and non-specific liver disease. A diagnosis of one of these risk factors significantly increased the risk of complications for cohort patients. The risk of cardiovascular disease was particularly increased in those clusters where both dyslipidemia and arterial hypertension were inclusion criteria (clusters 6–10). It was highest in cluster 10 (RR for AP: 10.10 CI: 9.28–10.98), where patients were also diagnosed with nicotine dependence. Patients in that cluster were also at increased risk of loss of extremities (RR: 4.21 CI: 1.5–11.84). The risk of stroke (RR: 4.32 CI: 3.95–4.71) and polyneuropathy (RR: 4.80 CI: 4.23–5.45) was highest in cluster 9, where patients were diagnosed with dyslipidemia, arterial hypertension, and depression, and also had a high prevalence of obesity. Cluster 6 (aHTN and dyslipidemia), cluster 7 (aHTN, dyslipidemia, and overweight/obesity), cluster 8 (aHTN, dyslipidemia and non-specific liver disease) and cluster 9 (aHTN, dyslipidemia and depression) represented cohorts with exaggerated risk of being diagnosed with cardiovascular disease (CVD) and kidney disease.

### Sex-Specific Relationships

**Figure 2:**
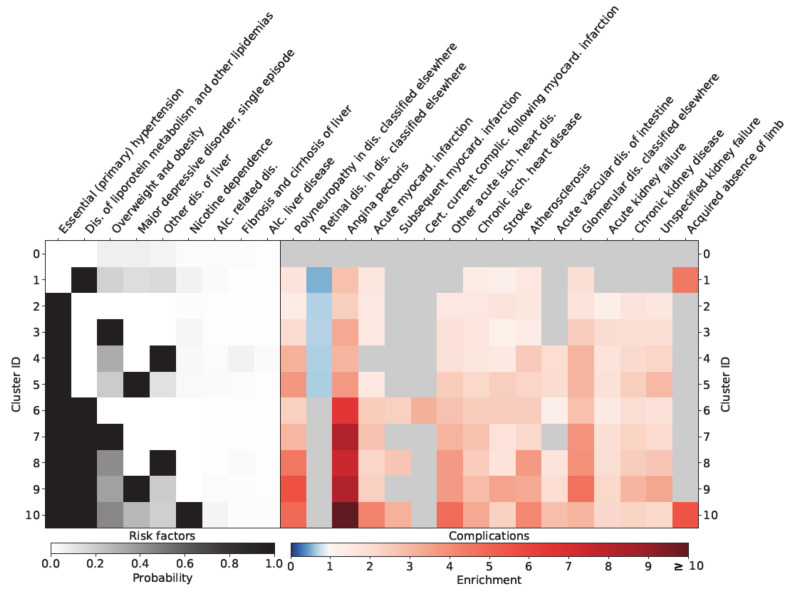
Relationship of different clusters with typical diabetes-specific complications in females.

**Figure 3:**
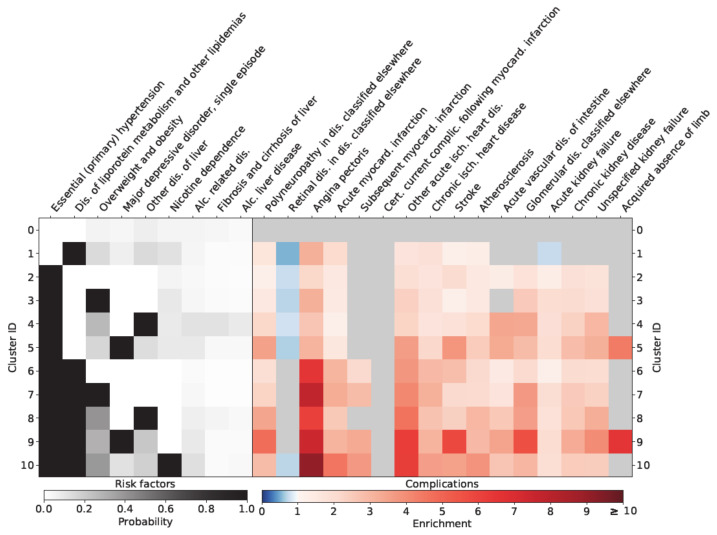
Relationship of different clusters with typical diabetes-specific complications in males.

As shown in Figure 2, females in cluster 6–10 were characterized by an exaggerated risk of being diagnosed with angina pectoris. In addition, the loss of extremities and angina pectoris (RR: 10.82 CI: 9.31–12.57) was extremely high for females in cluster 10 (aHTN, dyslipidemia and nicotine dependence). Similar results regarding angina pectoris could be observed for males in clusters 6–10 (Figure 3). Males in cluster 9 (aHTN, dyslipidemia and depression) additionally showed an exaggerated risk of CVD (RR for AP: 7.46 CI: 6.61–8.43), stroke (RR: 5.85 CI: 5.11–6.69), kidney disease (RR for chronic kidney disease: 3.28 CI: 3.06–3.53), and loss of extremities.

## 4. Discussion

Exploiting a cross-sectional clustering approach, in this study, we presented ten different risk phenotypes of diabetes mellitus (given our cohort age, these patients will be diagnosed predominately by diabetes mellitus type 2) based on commonly associated risk factors that allow for risk stratification for 16 typical diabetes complications. The combination of arterial hypertension and dyslipidemia was related to an increased risk of being diagnosed with diabetic complications. The risk of being diagnosed with micro- and macrovascular complications was particularly increased if an additional diagnosis of depression, nicotine dependence, obesity, or non-specific liver disease was present. In females, especially clusters 9 (aHTN, dyslipidemia and depression) and 10 (aHTN, dyslipidemia, and nicotine dependence) and in males, cluster 9 represents patients with a severely increased risk of being diagnosed with multiple diabetes complications.

In recent decades, evidence from large trials has greatly refined the therapeutic approach for diabetic patients. Data from the UK Prospective Diabetes Study (UKPDS) highlighted the central role of glycemic control in the prevention of diabetes-associated complications, showing, among other things, a risk reduction of 25% for microvascular disease upon intensified glucose-lowering therapy [23] and a 32% reduction for any diabetes-related endpoint for overweight diabetics receiving metformin [24]. Furthermore, additional factors such as cholesterol levels or blood pressure have been shown to influence macrovascular outcomes of patients with diabetes mellitus in the CARDS trial, a randomized controlled trial assessing the effect of an add-on low dose of atorvastatin therapy in type 2 diabetics [5], and the ADVANCE trial, which investigated the effect of a fixed angiotensin converting enzyme (ACE) inhibitor–diuretic combination in type 2 diabetics irrespective of baseline blood pressure [4]. Following up on this, more recent studies have identified several risk factors that increase the risk of micro- and macrovascular complications in diabetic patients [8,10,11]. Diagnoses such as arterial hypertension and dyslipidemia, in particular, are related to major macrovascular complications such as coronary artery disease or stroke [25,26] and microvascular complications like diabetic retinopathy or renal disease [27]. However, the risk of developing micro- and macrovascular diseases as a function of different combinations of risk factors is an important topic in type 2 diabetic patients, as diabetic patients often present with more than one comorbidity [28,29]. In the present study, we cross-sectionally identified five clusters of diabetic patients with different combinations of common comorbidities of diabetes, each of them showing an exaggerated risk of being diagnosed with micro- and macrovascular diabetic complications. These five clusters share the diagnoses of dyslipidemia and arterial hypertension as inclusion criteria, alongside combinations of smoking, depression, obesity, or non-specific liver disease. An increased risk of being diagnosed with micro- and macrovascular complications could be observed in the subgroup of diabetic patients with arterial hypertension and dyslipidemia, who also had a positive history of smoking. In this specific cluster, there was an increased risk of being diagnosed with CVD, atherosclerosis, and amputation of the legs. In a sex-specific analysis, a close relationship between smoking and diagnosis of angina pectoris and amputation of the legs in women could be observed. Smoking has been associated with an increased risk of glycemic progression in diabetics [30] as well as with an increased risk of macrovascular complications, especially peripheral artery disease, coronary artery disease [31], and diabetic foot ulcerations findings [32], which are superimposable with the risk characteristics of subgroup “cluster 10”. The underrepresentation of affections of the retina in nicotine-dependent diabetic patients is in accordance with a study by Cai et al., who noted a decreased risk of diabetic retinopathy and proliferative diabetic retinopathy [33]. Nevertheless, our results highlight that the combination of arterial hypertension, dyslipidemia, and smoking defines a specific cohort at a major risk of being diagnosed with diabetic complications. Next to that, cluster 9, characterized by type 2 diabetic patients diagnosed with arterial hypertension, dyslipidemia, and additional depression, also showed a close relationship to macro- and microvascular disease. Hence, men in this cluster were particularly at increased risk, especially of leg amputations. To date, co-occurrence of depression and type 2 diabetes has been identified as a major risk factor for chronic kidney disease, coronary heart disease, stroke, and increased all-cause mortality in a retrospective analysis investigating 933,211 patients. Although patients with depression were younger and had a higher estimated glomerular filtration rate (eGFR) than diabetics without depression in the study by Novak et al., they were characterized by a higher rate of comorbidities [8]. Accordingly, in the present study, we could show that the combination of depression with aHTN and dyslipidemia is especially related to an increased risk of being diagnosed with typical diabetes-specific complications. One possible explanation for the elevated risk of complications in depressed diabetic patients could be deficient glycemic control, as patients with depression are characterized by a worse glycemic profile compared to type 2 diabetic patients without depression [34]. The third high-risk cluster in the present study, cluster 8, comprised type 2 diabetic patients with a predominant co-occurrence of aHTN and dyslipidemia additional to the diagnosis of non-specific liver disease such as non-alcoholic fatty liver disease (NAFLD). NAFLD is in general closely related to metabolic disorders like diabetes mellitus and the respective complications. Evidence points to a crosstalk between inflamed abdominal fatty tissue and the liver, combined with a state of gut microbial dysbiosis accompanied by intestinal barrier dysfunction, leading to systemic inflammation, which consequently constitutes a driving factor in the development of vascular complication in diabetic patients [35]. Cluster 7 is defined by type 2 diabetic patients with arterial hypertension, dyslipidemia, and obesity. To date, several cluster analyses have reported an increased risk of diabetes-specific complications in type 2 diabetic patients suffering from obesity [13,36].

In this study, we clustered cross-sectionally, and hence one limitation is that causal relationships cannot be evaluated. A further limitation of the present study is that we did not have access to 4-point ICD codes that would have allowed analysis of the combinations of the underlying diseases with comorbidities. Nor did we have access to data on laboratory parameters, anthropometric parameters, or prescribed medications. A further limitation is that we defined our cohort with the ICD-codes E10–E14, which also includes diagnosis of diabetes mellitus type 1. However, given our cohort age, the diagnoses of the patients in the present study will be dominated by patients with diabetes mellitus type 2. Additionally, we cannot draw conclusions as to whether adequate diabetes therapy is related to changes in the risk of complications. Another limitation is that we had no access to data on lifestyle habits such as physical activity and nutritional data, which are closely related to the development of insulin resistance and diabetes mellitus [37].

## 5. Conclusions

In conclusion, our results demonstrate that the combination of arterial hypertension and dyslipidemia is especially associated with an elevated risk of diabetes-associated complications. Furthermore, an additional diagnosis of one of the following diseases, namely depression, obesity, non-specific liver disease, or the habit of smoking dramatically aggravates the risk of being diagnosed with micro- or macrovascular diseases in a diabetic patient collective. Therefore, patients who present with the unfavorable combinations of risk factors defined by these five clusters should be screened regularly and an intensified treatment and observation regimen may be implemented in the management algorithm of type 2 diabetes mellitus. In particular, the increasing prevalence of smoking in women in recent years [38] calls for better smoking cessation programs. Additionally, the close relationship between depression and typical diabetic complications already demonstrated by earlier work and shown to be even more pronounced in males in the present study calls for regular screening programs for depression in all diabetic patients, allowing for establishment of appropriate treatment [39]. However, prospective clinical studies would be necessary to further investigate the relationship between different risk factor combinations and the development of diabetes-specific outcomes.

## Figures and Tables

**Table 1:** Descriptive characteristics of the study cohort.

Cohort Size	195,575
Ratio of Males	50,3%
Minimum Age of Patients in 2014	60 y
Median Age Males in 2014 (Lower-Upper Quartile)	74 (67–82) y
Median Age Females in 2014 (Lower-Upper Quartile)	82 (73–89) y

**Table 2:** Numbers of individuals included in the different clusters. The reported ages refer to the end of the observation period (2014). The values in brackets give lower and upper quartiles, respectively.

Cluster ID	Number of Patients	Female Ratio	Mean Age Females/y	Mean Age Males/y
0	37,899	0.47	81 (72–91)	75 (66–83)
1	7124	0.42	76 (67–84)	71 (64–77)
2	63,518	0.52	83 (75–91)	77 (69–84)
3	10,600	0.54	76 (68–83)	72 (65–77)
4	6844	0.45	79 (71–87)	74 (67–81)
5	9057	0.70	82 (75–90)	78 (69–86)
6	29,621	0.45	80 (74–88)	75 (68–81)
7	10,399	0.48	76 (69–82)	72 (66–77)
8	6740	0.43	77 (70–84)	73 (67–78)
9	7778	0.68	80 (73–88)	75 (68–82)
10	5995	0.27	72 (65–76)	70 (64–74)

## Data Availability

Data were provided by the Federal Ministry for Health of Austria.

## Supplementary Materials

Supplementary information is available online at https://www.mdpi.com/article/10.3390/jpm11050328/s1.
