# Supplementary material for: Risk of Typical Diabetes-Associated Complications in Different Clusters of Diabetic Patients: Analysis of Nine Risk Factors
Source: J Pers Med. 2021 Apr 22;11(5):328. doi: 10.3390/jpm11050328 (PMC8143487; doi:10.3390/jpm11050328)
Supplement: Supplementary file 1 [file jpm-11-00328-s001.zip › jpm-1169414-supplementary.pdf]

Supplementary information to "Risk of typical diabetes-associated complications in different clusters of type 2 diabetic patients: cluster analysis of ten risk factors"

November 12, 2020

1 Clustering algorithm

The clustering algorithm used in this work is a modified version of the algorithm DIVCLUS-T [1], which was also applied and explained in the supplementary material of [2].

The input data passed to the DIVCLUS-T algorithm takes the form of a matrix $\mathbf{X}$ with M rows and N columns. Each row corresponds to one observation and each column corresponds to one feature, and the entry in the kth row and ith column of $\mathbf{X}$ is one if observation k has feature i and 0 else. In our case, each observation corresponds to one patient of the cohort, and each column corresponds to one medical diagnosis, and the entry of $\mathbf{X}$ in the kth row and ith column of $\mathbf{X}$ is one if patient k has been diagnosed with disease i and zero else. Starting from the root node, the DIVCLUS-T algorithm iteratively constructs a binary clustering tree, where each node represents a subset of all observations. The subsets represented by all leave nodes of the tree form a partition of the set of observations and are called clusters.

Let at any point during the run of the clustering algorithm a given node be a leaf of the clustering tree constructed thus far, and let S be the set of observations corresponding to it. For $1 \leq i \leq N$, denote by p_i the probability that the feature i is present in a randomly selected observation belonging to S. The inertia of the node is defined as

$$I = |S| \, \langle \mathbf{w}, \mathbf{p} \circ (1 - \mathbf{p}) \rangle, \tag{1}$$

where $\mathbf{p} = (p_1, p_2, \ldots, p_N)$, and $\circ$ and $\langle \, , \rangle$ denote the Hadamard and standard scalar product, respectively. The weight vector $\mathbf{w} = (w_1, \ldots, w_N)$ has entries between 0 and 1 and can be used to put different importance on different features. The child nodes of node k are defined by the presence, respectively the absence of one feature j $(1 \leq j \leq N)$, meaning that one of the child nodes corresponds to the subset $S_0^{(j)}$ of S of observations which do not have feature j, and the other one corresponds to the subset $S_1^{(j)}$ of S of observations which do have feature j. The feature j is chosen as

$$j = \underset{s}{\operatorname{argmin}} \left\{ I_0^{(s)} + I_1^{(s)} \middle| 1 \leq s \leq N \wedge v_s = 1 \right\}, \tag{2}$$

where $I_0^{(s)}$ and $I_1^{(s)}$ are the inertiae of the child nodes defined by the absence, respectively presence of feature s. The binary row vector $\mathbf{v} = (v_1, v_2, \ldots, v_N)$ specifies which features are allowed to be used for splitting a node.

Here we modify the definition of the inertia in Eq. (2) as follows. In Eq. (2), we replace the $I_k^{(s)}$ for $k = 0, 1$ by

$$\widehat{I_k}^{(s)} = |S_k^{(s)}| \sum_{i=0}^{N} w_i \, (p_{k,i}^{(s)})^{\alpha_i} (1 - p_{k,i}^{(s)}), \tag{3}$$

where $1 \leq i \leq N$, and $p_{k,i}^{s}$ denotes the probability that a randomly selected observation from $S_k^{(s)}$ has feature i. The exponents are defined as

$$\alpha_i = \frac{p_i}{1 - p_i}, \tag{4}$$

with p_i as above.

We choose the weight vector $\mathbf{w}$ such that uniform weight is given to all complications, while zero weight is given to all risk factors. The vector $\mathbf{v}$, which defines the set of allowed features used to split a node is chosen to be 1 for features corresponding to risk factors and 0 for those corresponding to complications. This choice means that we are clustering patients according to their risk factors, to maximize the difference in terms of complications between the clusters. To maintain sufficient statistical power, we stop splitting nodes if one of the resulting child nodes would represent less than 5000 observations.

References

[1] M. Chavent, Y. Lechevallier, and O. Briant, "DIVCLUS-T: A monothetic divisive hierarchical clustering method," *Computational Statistics and Data Analysis*, vol. 52, no. 2, pp. 687–701, 2007.

[2] N. Haug, C. Deischinger, M. Gyimesi, A. Kautzky-Willer, S. Thurner, and P. Klimek, "High-risk multimorbidity patterns on the road to cardiovascular mortality," *BMC Medicine*, vol. 18, no. 1, pp. 1–12, 2020.
